# Coupling the modules of EMT and stemness: A tunable ‘stemness window’ model

**DOI:** 10.18632/oncotarget.4629

**Published:** 2015-07-23

**Authors:** Mohit Kumar Jolly, Dongya Jia, Marcelo Boareto, Sendurai A. Mani, Kenneth J. Pienta, Eshel Ben-Jacob, Herbert Levine

**Affiliations:** ^1^ Center for Theoretical Biological Physics, Rice University, Houston, TX 77005-1827, USA; ^2^ Graduate Program in Systems, Synthetic and Physical Biology, Rice University, Houston, TX 77005-1827, USA; ^3^ Department of Bioengineering, Rice University, Houston, TX 77005-1827, USA; ^4^ Department of Physics and Astronomy, Rice University, Houston, TX 77005-1827, USA; ^5^ Department of Biosciences, Rice University, Houston, TX 77005-1827, USA; ^6^ School of Physics and Astronomy and The Sagol School of Neuroscience, Tel-Aviv University, Tel-Aviv 69978, Israel; ^7^ Institute of Physics, University of Sao Paulo, Sao Paulo 05508, Brazil; ^8^ Department of Translational Molecular Pathology, and Metastasis Research Center, The University of Texas MD Anderson Cancer Center, Houston, TX 77030, USA; ^9^ The James Brady Urological Institute, Johns Hopkins School of Medicine, Baltimore, MD 21287, USA; ^10^ Departments of Urology, Johns Hopkins School of Medicine, Baltimore, MD 21287, USA; ^11^ Department of Oncology, Johns Hopkins School of Medicine, Baltimore, MD 21287, USA; ^12^ Department of Pharmacology and Molecular Sciences, Johns Hopkins School of Medicine, Baltimore, MD 21287, USA

**Keywords:** partial EMT, stemness window, cancer stem cells, OVOL, multistability

## Abstract

Metastasis of carcinoma involves migration of tumor cells to distant organs and initiate secondary tumors. Migration requires a complete or partial Epithelial-to-Mesenchymal Transition (EMT), and tumor-initiation requires cells possessing stemness. Epithelial cells (E) undergoing a complete EMT to become mesenchymal (M) have been suggested to be more likely to possess stemness. However, recent studies suggest that stemness can also be associated with cells undergoing a partial EMT (hybrid E/M phenotype). Therefore, the correlation between EMT and stemness remains elusive. Here, using a theoretical framework that couples the core EMT and stemness modules (miR-200/ZEB and LIN28/let-7), we demonstrate that the positioning of ‘stemness window’ on the ‘EMT axis’ need not be universal; rather it can be fine-tuned. Particularly, we present OVOL as an example of a modulating factor that, due to its coupling with miR-200/ZEB/LIN28/let-7 circuit, fine-tunes the EMT-stemness interplay. Coupling OVOL can inhibit the stemness likelihood of M and elevate that of the hybrid E/M (partial EMT) phenotype, thereby pulling the ‘stemness window’ away from the M end of ‘EMT axis’. Our results unify various apparently contradictory experimental findings regarding the interconnection between EMT and stemness, corroborate the emerging notion that partial EMT associates with stemness, and offer new testable predictions.

## INTRODUCTION

Metastasis and tumor relapse remain clinically insurmountable and claim more than 90% of cancer-related deaths [1]. It is believed that metastasis begins when some cancer cells of the primary tumor undergo an Epithelial-to-Mesenchymal Transition (EMT) and migrate towards blood vessels. These metastatic cells transit in the bloodstream as Circulating Tumor Cells (CTCs) and then exit at distant organs. There they may undergo the reverse of EMT, a Mesenchymal-to-Epithelial Transition (MET) and grow into secondary tumors [2]. On the other hand, tumor relapse is thought to be caused by therapy-resistant Cancer Stem Cells (CSCs) that can repopulate a tumor. Previous experimental studies have shown that these cell-fate decisions of phenotypic transition (EMT/MET) and gaining stem cell properties (stemness) are interconnected via underlying gene regulatory networks [3]. However, the basic principles of this interconnection remain enigmatic, hence limiting major therapeutic advances.

The decisions of EMT/MET and attaining stemness are much more flexible than imagined earlier—neither is EMT/MET a binary process nor is stemness a fixed inherent trait of a few cells. Cells undergoing EMT/MET can attain a hybrid epithelial/mesenchymal (E/M) phenotype that has combined epithelial (cell-cell adhesion) and mesenchymal (motility) traits. These combined traits enable them to migrate collectively, as observed in cluster migration of CTCs in the lung, prostate and breast cancer patients [4–7]. Also, CSCs and non-CSCs can interconvert and maintain a dynamic equilibrium among themselves [8–11]. Such plasticity blurs the direct one-to-one correlation between a complete EMT and an increased likelihood of being a CSC as postulated in earlier work [12–14]. As we will discuss below, this blurring can allow hybrid E/M (partial EMT) as well as epithelial cells to gain stemness, as observed in recent experimental studies [15–20].

**Figure 1:**
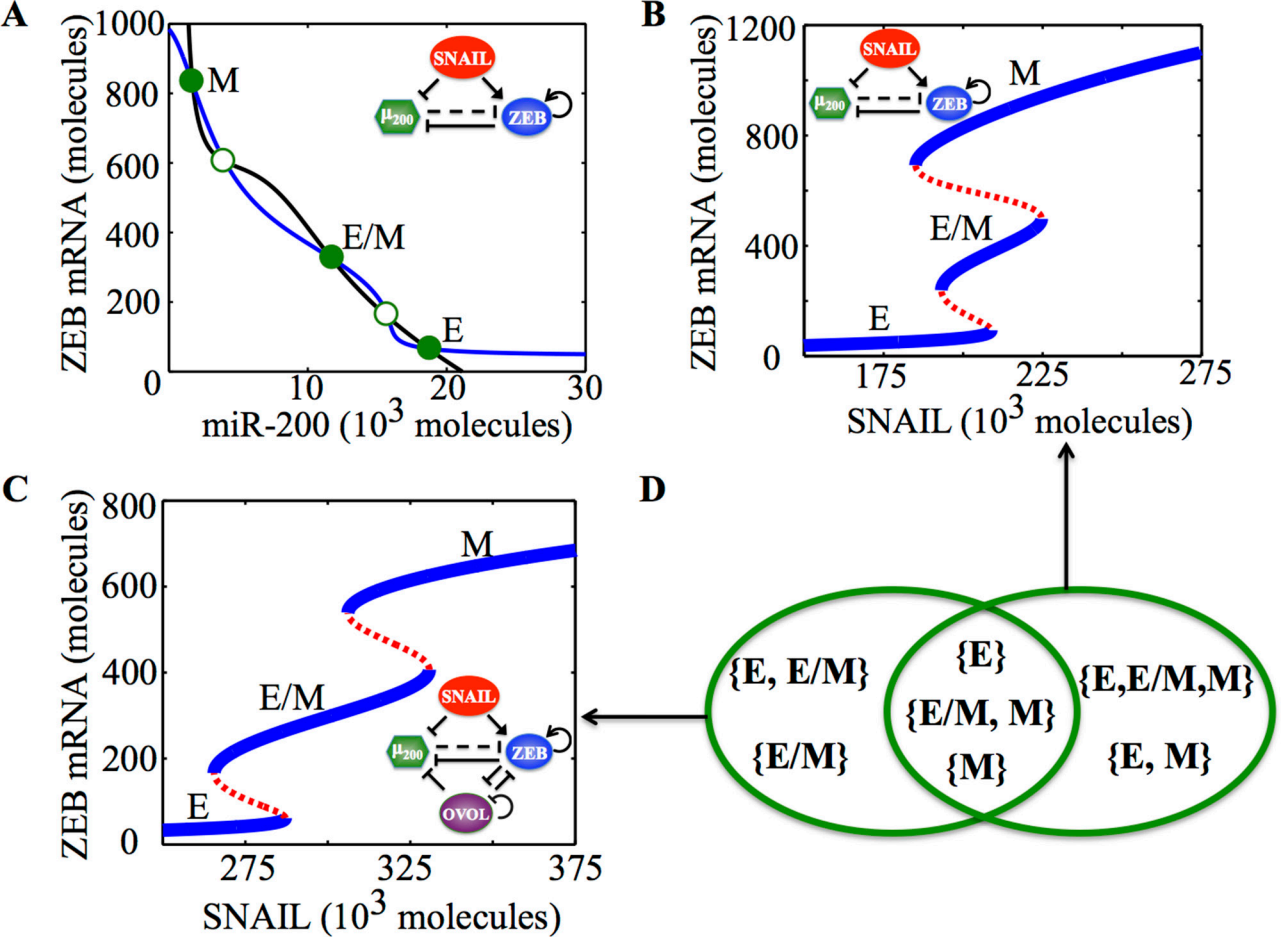
Dynamical system characteristics of the EMT decision-making circuit - (miR-200/ZEB) **A.** Nullclines of (miR-200/ZEB) circuit for SNAIL = 200 * 10^3^ molecules representing three steady states: Epithelial (E—high miR-200, low ZEB), hybrid Epithelial/Mesenchymal (E/M—medium miR-200, medium ZEB) and Mesenchymal (low miR-200, high ZEB). Solid green dots represent stable states; hollow green dots represent unstable states. **B.** Bifurcation levels of mRNA levels of ZEB in response to SNAIL for miR-200/ZEB circuit. **C.** Bifurcation levels of mRNA levels of ZEB in response to SNAIL for miR-200/ZEB/OVOL circuit. Different phenotypes obtained at different levels of SNAIL are labeled alongside. **D.** Comparing the different phases (co-existence of different phenotypes) obtained for miR-200/ZEB in (B) and miR-200/ZEB/OVOL circuits in (C).

Deciphering the EMT-stemness interplay requires a rigorous analysis of the decision-making modules of EMT and stemness, and the coupling between them. EMT decision-making is governed by a mutually inhibitory feedback loop of miR-200/ZEB [21, 22], such that epithelial cells have (high miR-200, low ZEB), mesenchymal cells have (low miR-200, high ZEB) and hybrid E/M cells have (medium miR-200, medium ZEB) [23, 24] (Figure 1A, Figure S1). On the other hand, stemness is regulated by a mutually inhibitory loop of LIN28/let-7 [25] that can allow three states - D (Down - low LIN28, high let-7), U (Up - high LIN28, low let-7) and D/U (Down/Up - medium LIN28, medium let-7) [18]. LIN28 activates the pluripotency marker OCT4 [26]. Both very high and very low levels of OCT4 lead to loss of stem cell properties (stemness), therefore OCT4 levels must be within a range of intermediate levels to acquire or maintain stemness [27–30]. Such intermediate levels of OCT4 are generally attained by medium levels of LIN28 or equivalently D/U state, therefore, the D/U state usually associates with stemness [18]. Consequently, the phenotype(s)—E (no EMT), M (complete EMT), and E/M (partial EMT)—that can have medium OCT4 (or corresponding LIN28) levels can gain stemness, or in other words, lie in a ‘stemness window’.

A key player that has been reported independently both in regulating EMT and stemness, and is therefore, well-positioned to tune the EMT-stemness interplay is the transcription factor OVOL. OVOL is a well-studied regulator of embryogenesis that is involved in the differentiation of epidermal progenitor cells [31, 32] and is self-inhibitory [33]. In a developmental example of EMT—the case of mammary morphogenesis—OVOL is expressed in terminal end bud (TEB) cells that migrate collectively forming finger-like projections [34]. It can maintain TEB cells in a hybrid E/M phenotype and operate as a ‘critical molecular brake on EMT’ by preventing the ‘TEB cells that have gained partial plasticity’ from undergoing complete EMT [34]. Also, in a pathological EMT context, OVOL can drive MET by forming a double negative feedback loop with ZEB [35]. Collectively, coupling miR-200/ZEB with OVOL significantly expands the range of parameters or physiological conditions for the existence of the hybrid E/M phenotype—such that without OVOL, E/M phenotype can only exist in combination with the mesenchymal phenotype (the possible phases are {E, E/M, M} and {E/M, M}), however, with OVOL, the E/M phenotype can either exist alone or co-exist with the epithelial phenotype (phases {E/M} and E, E/M}) (Figure 1B–1D, ref. [36]). However, the mechanism by which OVOL influences the EMT-stemness interplay remains elusive.

Here, we present a theoretical approach to investigate how OVOL modulates EMT-stemness interplay. First, we investigate the coupled dynamics of EMT and stemness circuits—(miR-200/ZEB) and (LIN28/let-7) respectively—to elucidate how different relative strengths of the two links coupling these two circuits—miR-200 inhibiting LIN28 (referred to as ‘feed-forward coupling’ hereafter) and let-7 inhibiting ZEB (referred to as ‘feed-backward coupling’ hereafter)—affect EMT-stemness interplay. Further, we couple OVOL to this combined network, and compare the EMT-stemness correlation obtained with that obtained for the combined network without OVOL. We find that OVOL can enable cells in the epithelial and hybrid E/M states, but not the mesenchymal one, to gain stemness, i.e. it prevents the ‘stemness window’ from sliding completely towards the M end of the axis. Consequently, inhibition or loss of OVOL can allow the cells that undergo a complete EMT (M phenotype) to gain stemness. Our study serves two purposes—it presents an example of how various coupling factors may influence the EMT-stemness correlation, and it highlights the specific effects of OVOL as an example of a modulating factor.

## RESULTS

### Coupling the decision-making modules of EMT and stemness

**Figure 2:**
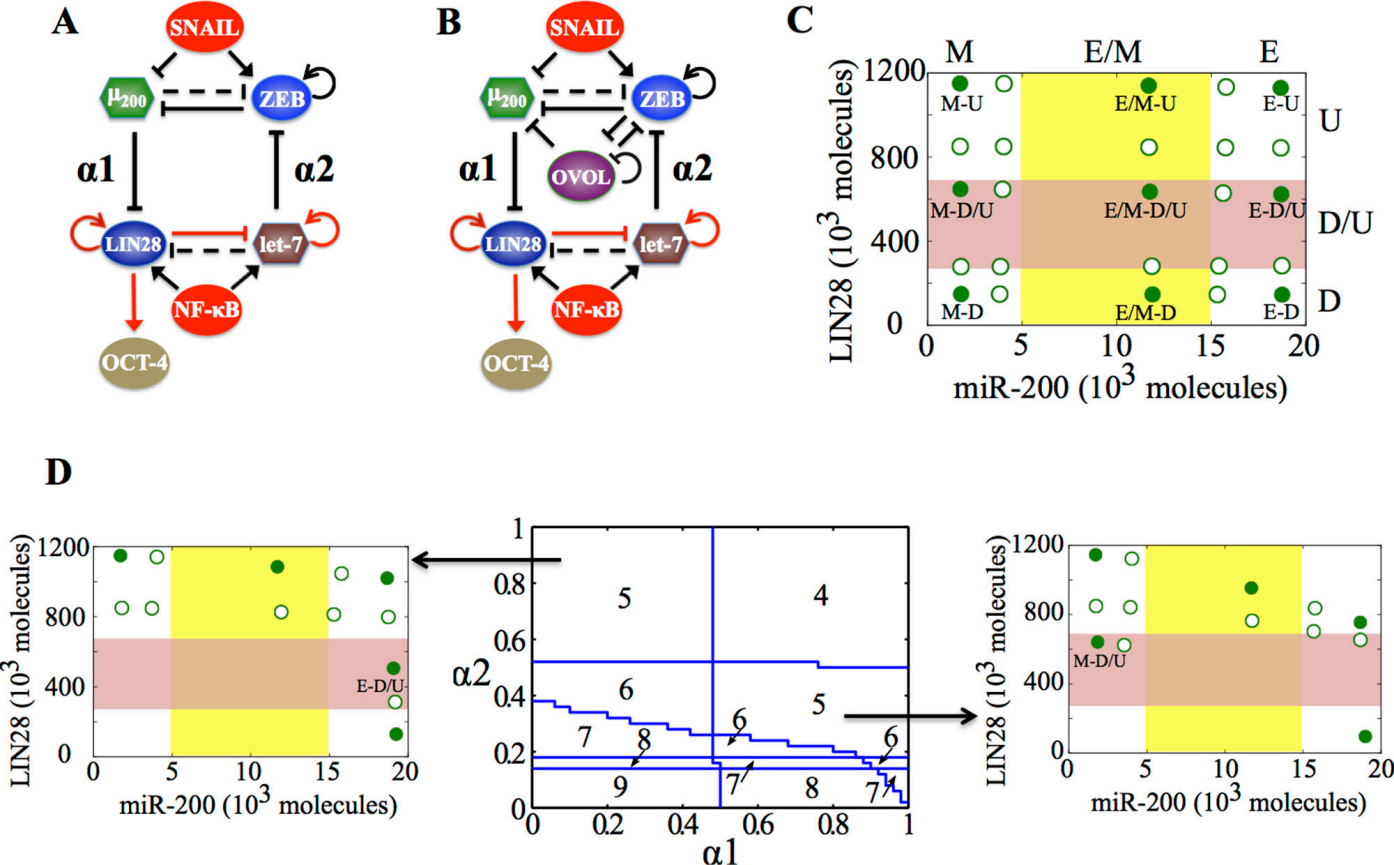
Coupling the decision-making circuits for EMT and stemness **A.** Regulatory network showing coupling of miR-200/ZEB circuit with LIN28/let-7 circuit. **B.** Regulatory network showing coupling of miR-200/ZEB/LIN28/let-7 circuit with the transcription factor OVOL. A black solid arrow denotes transcriptional activation, and a black solid bar denotes transcriptional inhibition. Black dashed line indicates microRNA-mediated regulation, and red solid arrows imply non-transcriptional activation. α1 and α2 are coupling parameters between the EMT circuit (miR-200/ZEB) and the stemness circuit (LIN28/let-7) that vary from 0 to 1. The larger the value of α1, the stronger the inhibition of LIN28 by miR-200; and the larger the value of α2, the stronger the inhibition of ZEB by let-7. **C.** Phenotypic map representing all possible steady states of the coupled circuit (miR-200/ZEB/LIN28/let-7) for SNAIL = 200 * 10^3^ molecules and NF-kB = 25 * 10^3^ molecules, at α1 = α2 = 0.05 (very weak ‘feed-forward’ and ‘feed-backward’ coupling). Red shaded region defines the ‘stemness window’ based on relative LIN28 levels or equivalently relative OCT4 levels, and yellow shaded region represents range of miR-200 levels for the existence of the hybrid E/M phenotype, as noted in [23] for (miR-200/ZEB) circuit. **D.** *(center)* Phase diagram representing the number of stable steady states of the coupled circuit (miR-200/ZEB/LIN28/let-7) for varying values of (α1, α2) at SNAIL = 200 * 10^3^, and NF-kB = 25 * 10^3^ molecules. *(left)* Stable states of the coupled circuit at α1 = 0.3 and α2 = 0.9. *(right)* Stable states of the coupled circuit at α1 = 0.9 and α2 = 0.3. Green solid dots represent stable states, and green hollow dots are for unstable states. The labels ‘E-D/U’ (*left*) and ‘M-D/U’ (*right*) denote that epithelial and mesenchymal phenotypes respectively lie in stemness window.

The EMT and stemness decision-making modules—(miR-200/ZEB) and (LIN28/let-7)—are driven by SNAIL and NF-kB respectively (Figure 2A, 2B). These inputs govern which phenotypes or states a cell can adopt, even in the absence of any coupling. These modules are connected via two links: miR-200 inhibiting LIN28 (‘feed-forward coupling’) and let-7 inhibiting ZEB (‘feed-backward coupling’). The strengths of these couplings are represented by variables α1 and α2. Previously, we demonstrated that in the absence of any ‘feed-backward coupling’ (inhibition of ZEB by let-7), both the hybrid E/M and M phenotypes can gain stemness, with the hybrid E/M being more likely to do so [18]. As the first step to decipher the full EMT-stemness interplay, we include here the ‘feed-backward coupling’ from let-7 to ZEB and calculate the total number of stable steady states of the combined network (miR-200/ZEB/LIN28/let-7) at different values of (α1, α2).

We start with the case when both (miR-200/ZEB) and (LIN28/let-7) are stand-alone (i.e. without any coupling, α1 = α2 = 0) tristable circuits, i.e. the input parameters are set such that cells can attain any of the three phenotypes—E, E/M and M—and each of them independently can be associated with any of the three states of the LIN28/let-7 circuit—D, D/U, and U—thus leading to a total of 3 * 3=9 stable states. Each of these stable states must be defined by a set of two variables, for example, the levels of LIN28 and levels of miR-200, as shown in the phenotypic map (Figure 2C). As either the ‘feed-forward coupling’ or ‘feed-backward coupling’ gets stronger, or in other words, as either α1 (strength of inhibition of LIN28 by miR-200) or α2 (strength of inhibition of ZEB by let-7) increases, the number of total stable states decreases, indicating that some stable states can coalesce at different coupling strengths (α1, α2) (Figure 2D, middle).

We found that even when the total number of stable states of the coupled circuit (miR-200/ZEB/LIN28/let-7) is the same, different phenotype(s) can gain stemness, depending on exact values of strengths of ‘feed-forward coupling’ and ‘feed-backward coupling’, or equivalently (α1, α2) (Figure 2D, left, right). The likelihood of gaining stemness of a phenotype is defined on the basis of relative OCT4 levels (relative to the saturation level of OCT4 when it is activated by a threshold level of LIN28). If that relative OCT4 level is between 0.25–0.65, the corresponding phenotype is highly likely to gain stemness or in other words, it lies in the ‘stemness window’. This definition of a ‘stemness window’ is based on experimental observations that both too high and too low levels of OCT4 can lead the cell to differentiate, hence medium OCT4 levels (usually corresponding to D/U [18]) correspond to pluripotency or stemness [27–30]. This range of OCT4 levels defining the ‘stemness window’ can be tumor-specific. The results presented here are for this hypothesized range and serve to demonstrate the overall concept.

**Figure 3:**
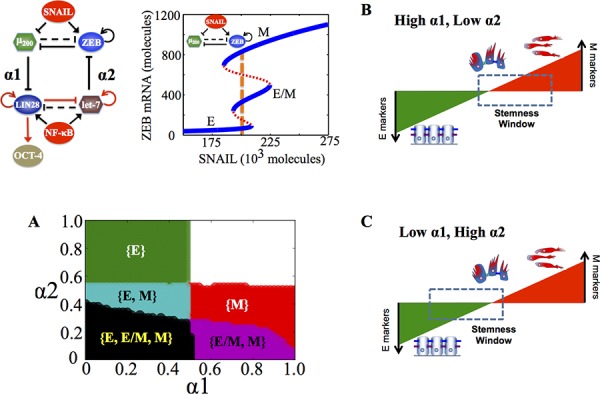
Stemness regions for different phenotypes under various (α1, α2), when cells are in {E, E/M, M} phase at α1 = α2 = 0 **A.** Phase diagram of the circuit representing the values of (α1, α2) for which the different phenotypes—E, M and E/M—can lie in stemness window respectively, for SNAIL = 200 * 10^3^ molecules and NF-kB = 25 * 10^3^ molecules. Areas with different colors represent different combinations of phenotypes than can gain stemness for that range of (α1, α2). **B, C.** Model for tunable ‘stemness window’ on the ‘EMT axis’, such that (high α1, low α2) allows for stemness to be associated with {E/M, M} and {M}, pushing the ‘stemness window’ towards the M end of the EMT axis, and (low α1, high α2) allows for stemness to be associated with {E}, pushing the ‘stemness window’ towards the E end of the EMT axis. The results presented here are for the circuit presented here, and SNAIL levels driving this circuit are marked by a dashed line in bifurcation diagram presented here.

To investigate further how ‘feed-forward coupling’ (miR-200 inhibiting LIN28) and ‘feed-backward coupling’ (let-7 inhibiting ZEB) affect which phenotypes—E, M and E/M—gain stemness, we calculated the stable states of the system at different values of (α1, α2) and plotted the ‘stemness region’ for each phenotype, i.e. range of values of (α1, α2) for which each phenotype can gain stemness. For some range of the values of (α1, α2), more than one phenotype can gain stemness, or in other words, the ‘stemness regions’ of the three phenotypes (E, M and E/M) can overlap among themselves. For instance, at low strengths of both ‘feed-forward coupling’ and ‘feed-backward coupling’ (low α1, low α2), all three phenotypes can gain stemness (black shaded region in Figure 3A), indicating that the ‘stemness window’ can cover the ‘EMT axis’, thereby enabling a rich phenotypic plasticity for cells that gain stemness. However, as either of the coupling strengths increase, this plasticity is restricted. Specifically, the epithelial (E) phenotype can be associated with stemness only at weak ‘feed-forward coupling’ (low α1), irrespective of the strength of ‘feed-backward coupling’ (α2) (green shaded region in Figure 3A). Conversely, the mesenchymal (M) phenotype can be associated with stemness only at weak ‘feed-backward coupling’ (low α2), irrespective of the strength of ‘feed-forward coupling’ (α1) (red and violet shaded regions in Figure 3A).

Therefore, the relative strength of these two coupling links govern exactly where the ‘stemness window’ lies on the ‘EMT axis’; strong ‘feed-forward coupling’ (high α1; strong inhibition of miR-200 on LIN28) push it towards the M end of the axis, while strong ‘feed-backward coupling’ (high α2; strong inhibition of let-7 on ZEB) push it towards the E end (Figure 3B, 3C). Our results reflect that the position of ‘stemness window’ on EMT axis is not universal, but rather coupling-dependent; hence cells in all three phenotypes—E, M and E/M—can possibly gain stemness depending on the relative levels of coupling strengths (α1, α2) between the EMT and stemness decision-making modules - (miR-200/ZEB) and (LIN28/let-7).

Next, we choose a different value of SNAIL such that at no coupling (α1 = α2 = 0), cells are all in the {M} phase, but can associate with any of the three states of (LIN28/let-7) circuit, i.e. hence a total 3 * 1 = 3 of stable states. Here, as α1 and α2 increase, total number of stable states increases, and again, this total number is not sufficient to determine which phenotype(s) correlate with stemness; as before, this correlation depends on the exact values of (α1, α2) (Figure S2, S3).

Generally, an increase or decrease in number of states shows that in general, increasing coupling between the two decision-making circuits; (miR-200/ZEB) and (LIN28/let-7) creates non-trivial associations between the states of EMT circuit and those of stemness circuit, therefore regulating which combination of phenotype(s) gain stemness. These results demonstrate that the set of phenotypes that can gain stemness, or in other words, the positioning of the ‘stemness window’ on the ‘EMT axis’, depends at least on two factors: (a) the relative strength of coupling links between EMT and stemness decision-making modules, and (b) the external input signals on EMT circuit, such as SNAIL that governs the different set of phenotypes a cell can attain—E, M, and E/M.

### OVOL precludes the mesenchymal phenotype from gaining stemness

Aside from the coupling strengths, it is expected that other factors may help determine the ‘context’ for the coupled EMT-stemness circuits. To show the effect of one such factor, we focus on the transcription factor OVOL and investigate how OVOL affects the correlation between EMT and stemness. To proceed, we analyze the coupled circuits without OVOL (miR-200/ZEB/LIN28/let-7) and with OVOL (miR-200 /ZEB/LIN28/let-7/OVOL) for varying values of SNAIL, such that without any coupling (α1 = α2 = 0), cells can attain either only the mesenchymal phenotype ({M}) or one of the two phenotypes: mesenchymal or partial EMT ({E/M, M}).

**Figure 4:**
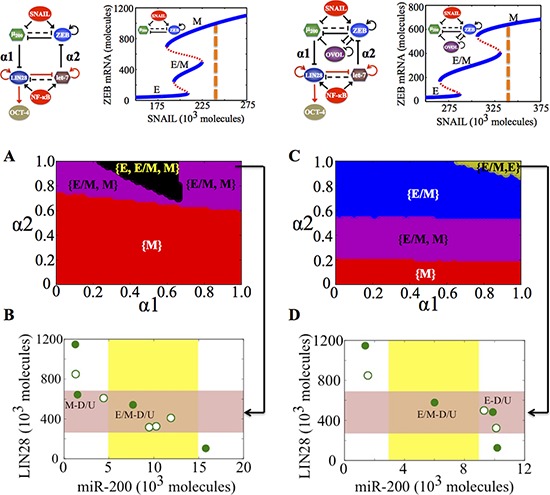
State-space characteristics of the coupled networks miR-200/ZEB/LIN28/let-7 and miR-200/ZEB/LIN28/let-7/OVOL, when cells are in {M} phase at α1 = α2 = 0 **A.** Phase diagram of the circuit miR-200/ZEB/LIN28/let-7 representing the values of (α1, α2) for which the different phenotypes can lie in stemness window, for SNAIL = 240 * 10^3^ molecules and NF-kB = 25 * 10^3^ molecules. **B.** Phenotypic map of the coupled circuit at α1 = α2 = 0.95 and at external signals SNAIL = 240 * 10^3^ molecules and NF-kB = 25 * 10^3^ molecules. Red shaded area shows the ‘stemness window’ based on relative OCT4 levels, and yellow shaded area represents the range of miR-200 levels for the existence of the hybrid E/M phenotype, as noted in [23] for (miR-200/ZEB) circuit and in [36] for (miR-200/ZEB/OVOL) circuit. **C, D.** represent a similar case for (A), (B) respectively but for the circuit with OVOL, therefore SNAIL = 340 * 10^3^ molecules. Different colors represent different combinations of phenotypes that can gain stemness. Steady state diagram and phase diagram in every column are for the circuit drawn in the topmost row of that column, such that at α1 = α2 = 0 (no coupling between the EMT and stemness circuits), cells are in the phase marked by dashed lines in the bifurcation diagram drawn next to the circuit.

We start with the case of high levels of SNAIL such that at α1 = α2 = 0, all cells are in a mesenchymal (M) phenotype and can gain stemness (monostable phase {M} at α1 = α2 = 0 in Figure 4A). We investigate the behavior at different values of α1 and α2, and plot ‘stemness regions’ for the phenotypes E, M, and E/M. In absence of OVOL, at a strong inhibition of ZEB by let-7 or ‘feed-backward coupling’ (α2 > 0.7), ZEB is suppressed strongly so as to allow the existence of the hybrid E/M phenotype in addition to the M phenotype and both of them lie in the ‘stemness window’ (Figure 4A, 4B). In the presence of OVOL, both these phenotypes—hybrid E/M and M—can gain stemness at a relatively weaker inhibition of ZEB by let-7 (α2 > 0.2), probably because OVOL is also inhibiting ZEB (Figure 4C). Further, at a strong ‘feed-backward coupling’ (α2 > 0.6), the M phenotype can no longer gain stemness, and only the hybrid E/M and E phenotypes lie in the ‘stemness window’ (Figure 4C, 4D). Also, at slightly higher levels of SNAIL, in the absence of OVOL, the only phenotype that lies in the stemness window is mesenchymal (M), but in presence of OVOL, the only phenotype that can gain stemness is the hybrid E/M phenotype (Figure S4). These results suggest that at strong ‘feed-backward coupling’, OVOL can drive the M phenotype out of the ‘stemness window’.

In the absence of OVOL, for a given range of α1 and α2, all the three phenotypes (E, M, and E/M) can gain stemness, representing a very flexible positioning of ‘stemness window’ all over the ‘EMT axis’. In other words, there can exist a rich phenotypic plasticity for the stem cells, as cellular stochastic fluctuations can cause motility transitions without necessarily interfering with a cell's stemness. In the presence of OVOL, however, this plasticity is significantly curtailed as there is no tristable {E, E/M, M} phase overlapping with the stemness region (compare the black area in Figure 4C vs that in Figure 4A).

**Figure 5:**
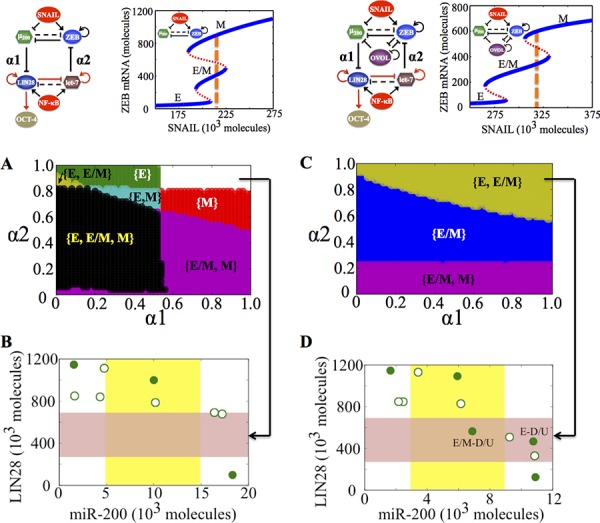
State-space characteristics of the coupled networks miR-200/ZEB/LIN28/let-7 and miR-200/ZEB/LIN28/let-7/OVOL, when cells are in {E/M, M} phase at α1 = α2 = 0 **A.** Phase diagram of the circuit miR-200/ZEB/LIN28/let-7 representing the values of (α1, α2) for which the different phenotypes can lie in stemness window, for SNAIL = 210 * 10^3^ molecules and NF-kB = 25 * 10^3^ molecules. **B.** Phenotypic map of the coupled circuit at α1 = α2 = 0.9 and at external signals SNAIL = 210 * 10^3^ molecules and NF-kB = 25 * 10^3^ molecules. Red shaded area shows the ‘stemness window’ based on relative OCT4 levels, and yellow shaded area represents the range of miR-200 levels for the existence of the hybrid E/M phenotype, as noted in [23] for (miR-200/ZEB) circuit and in [36] for (miR-200/ZEB/OVOL) circuit. **C, D.** represent a similar case for (A), (B) respectively but for the circuit with OVOL, therefore SNAIL = 320 * 10^3^ molecules. Different colors represent different combinations of phenotypes that can gain stemness. Steady state diagram and phase diagram in every column are for the circuit drawn in the topmost row of that column, such that at α1 = α2 = 0 (no coupling between the EMT and stemness circuits), cells are in the phase marked by dashed lines in the bifurcation diagram drawn next to the circuit.

A different value of SNAIL can be chosen, such that without coupling, cells can attain either a mesenchymal or partial EMT phenotype (the {E/M, M} phase at α1 = α2 = 0 in Figure 5A). In the absence of OVOL, a large range of values of (α1, α2) allows any of the three phenotypes (E, M, and E/M) to gain stemness, thus representing an enriched plasticity for stem cells as observed earlier too. However, in the presence of OVOL, this tristable phase {E, M, E/M} disappears (compare the black area in Figure 5C vs that in Figure 5A). Further, in absence of OVOL, none of the three phenotypes can lie in the stemness window at strong coupling (α1 = α2 = 1) (white area in Figure 5A), however, at the same values of α1 and α2, both hybrid E/M and E phenotypes can gain stemness after including OVOL (Figure 5A–5D).

Consistently, in stemness region diagrams, the presence of OVOL allows for a broad range of (α1, α2) values that enables an exclusive and exhaustive association of the hybrid E/M phenotype with stemness, a feature not observed for the circuit without OVOL (compare the blue area in Figure 5C vs that in Figure 5A). A significant stemness region also exists for the cells in the {E, E/M} phase, i.e. cells both in epithelial and hybrid E/M phenotype can gain stemness (Figure 5B). Collectively, these results suggest that OVOL restricts the sliding of ‘stemness window’ towards the M end of the ‘EMT axis’, or in other words, it typically precludes the mesenchymal phenotype from gaining stemness.

### Inhibition of OVOL allows mesenchymal phenotype to gain stemness

Next, we investigate the effect of OVOL in modulating EMT-stemness interplay, such that at no coupling (α1 = α2 = 0), all cells are in the hybrid E/M phenotype. Importantly, this {E/M} phase is allowed only when OVOL is coupled to miR-200/ZEB and is not present in the absence of OVOL (Figure 1D, ref. [36]). Therefore, instead of comparing our results for the combined circuit without OVOL, here we consider the effects of an external signal controlling OVOL so as to better understand its role.

**Figure 6:**
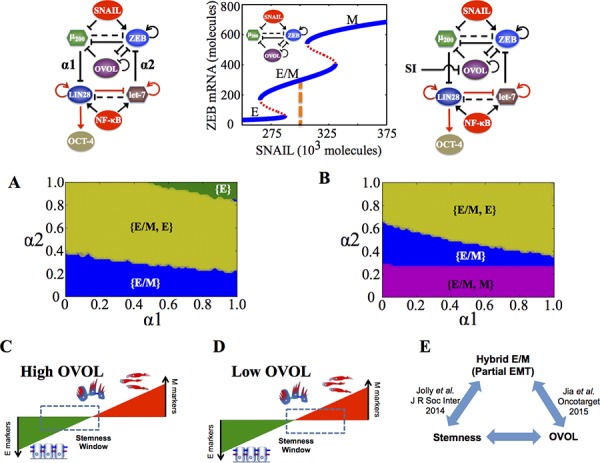
State-space characteristics of the miR-200/ZEB/let-7/OVOL circuit when the cells are in {E/M} phase at α1 = α2 = 0 **A, B.** Phase diagram of the circuit representing the values of (α1, α2) for which different phenotypes can attain stemness or lie in the ‘stemness window’, for SNAIL = 300 * 10^3^ molecules and NF-kB = 25 * 10^3^ molecules. (A) denotes the case without inhibition signal on OVOL (as shown in the circuit above (A)) and (B) is for the circuit with inhibition signal on OVOL (as shown in the circuit above (B)). Both (A, B) represent the case such that cells are in {E/M} phase at α1 = α2 = 0 (as shown by dashed line in the bifurcation figure). **C–D.** Flexible ‘stemness window’ model showing that (C) high levels of OVOL can pull the ‘stemness window’ towards the E end of the axis, and (D) low levels can let it slide towards the M end of the axis. **E.** Cartoon showing the interconnections between partial EMT or hybrid E/M phenotype, stemness and the effect of OVOL (Jolly et al). [18] suggest that hybrid E/M phenotype is enriched in stemness, Jia et al. [36] propose that endogenous OVOL levels can expand the range of existence of the hybrid E/M phenotype, and results here show that endogenous levels of OVOL can enable an association of stemness with the hybrid E/M phenotype.

As before, we explore the effect of different values of coupling strengths (α1, α2) on the EMT-stemness coupling, by calculating ‘stemness regions’ for all three phenotypes over the entire range of values of the coupling parameters (α1, α2). At weak ‘feed-backward coupling’ (low α2), only the hybrid E/M phenotype can gain stemness, however, as the strength of this coupling increases, ZEB is further repressed so as to allow the existence of the epithelial phenotype in addition to hybrid E/M phenotype, and both these phenotypes lie in the ‘stemness window’ (Figure 6A). At α1 = α2 = 1, only the E phenotype can gain stemness, i.e. the ‘stemness window’ moves completely to E end of the ‘EMT axis’ as the levels of ZEB are suppressed strongly by let-7 (very strong ‘feed-backward coupling’) (Figure 6A, 6C). Consistently, upon overexpression of OVOL (through an external activation signal), the E phenotype can lie in ‘stemness window’ for a broad range of coupling strengths (α1, α2) (Figure S5). Next, an external inhibition signal on OVOL is applied and the stemness regions for E, E/M, and M phenotypes are calculated. Inhibiting OVOL allows both M and E/M phenotypes to gain stemness without any coupling ({E/M, M} phase at α1 = α2 = 0 in Figure 6B) as well as for weak ‘feed-backward coupling’ (α2 < 0.2) irrespective of the strength of ‘feed-forward coupling’. Further, it reduces the range of (α1, α2) over which {E/M} and {E, E/M} phases can be associated with stemness, and enables the association of {E/M, M} phase with stemness (Figure 6B), i.e. inhibition of OVOL allows the ‘stemness window’ to slide towards the M end of the ‘EMT axis’, especially with the ‘feed-forward coupling’ being stronger than the ‘feed-backward coupling’ (low α2) (Figure 6C, 6D).

In summary, at a strong ‘feed-backward coupling’ and in presence of endogenous levels of OVOL, the M phenotype is precluded from gaining stemness. Consistently, if either OVOL is inhibited or the ‘feed-backward coupling’ (inhibition of ZEB by let-7) is weakened, ZEB levels can be maintained at high levels due to its self-activation, hence enabling the M phenotype as well as increasing its likelihood to gain stemness. Therefore, OVOL and the inhibition of ZEB by let-7 can act synergistically to preclude the complete EMT (or mesenchymal) phenotype from gaining stemness, therefore indicating a possible decoupling between complete EMT and stemness.

## DISCUSSION

Coupling between EMT and stemness (or tumor-initiating properties) directly connects two of the most fatal aspects of cancer—metastasis and tumor relapse. The emergence of stemness when cells undergo a complete EMT has been studied at a phenomenological level [13, 14]; however, an understanding of how the underlying decision-making modules couple these traits together has largely remained underexplored. Here, using a theoretical framework modeling the connections between the decision-making modules of EMT (miR-200/ZEB) and stemness (LIN28/let-7), we reveal how the strengths of the two links between these modules—inhibition of LIN28 by miR-200 (‘feed-forward coupling’) and inhibition of ZEB by let-7 (‘feed-backward coupling’)—determine which phenotype(s) gain stemness, or in other words, where the ‘stemness window’ lies on the ‘EMT axis’. We also investigate the role of OVOL, a transcription factor noted for its roles in regulating stemness and shepherding EMT/MET [31–39].

We find that relative strengths of ‘feed-forward coupling’ and ‘feed-backward coupling’ can affect the position of ‘stemness window’ on the ‘EMT axis’—strong inhibition of LIN28 by miR-200 pushes it towards mesenchymal (M) end of the axis, but strong inhibition of ZEB by let-7 pushes it towards the E end. miR-200 family that is highly expressed in epithelial phenotype [21, 22], has been experimentally shown to inhibit the gain of stemness [40]. However, the predicted role of let-7 needs further investigation.

We further show that OVOL modulates the positioning of the ‘stemness window’ on the ‘EMT axis’ in multiple ways: (a) it precludes the association of an entirely mesenchymal phenotype with stemness, (b) it significantly increases the likelihood for the hybrid E/M phenotype to gain stemness, and (c) it restricts the range of physiological conditions (or parameters) under which minor cellular stochastic fluctuations can cause motility transitions in stem cells, or in other words, it prevents the ‘stemness window’ from sliding all over the ‘EMT axis’. These results explain why the loss of OVOL may result in unchecked or aberrant plasticity of stem or progenitor cells [34, 41]. In developmental contexts, this unrestricted plasticity can impair the differentiation potential of stem or progenitor cells [41]; however, higher plasticity might be advantageous for tumor progression by potentially maximizing the range of conditions under which cells can gain stemness. Therefore, such enriched plasticity is more likely to be a hallmark of cancer but not necessarily that of homeostasis or development, thus offering some insight into possible differences in EMT during cancer metastasis (type III EMT) vs. EMT during embryonic development and tissue regeneration (type I, II EMT). This observation also offers a possible explanation of why the loss of OVOL correlates with poor survival [35].

Further, our results reveal that OVOL can not only ‘maintain’ the hybrid E/M phenotype [34, 36], but also largely associate it with stemness (Figure 6E). Based on its proposed effect in enabling collective migration of tumor cell clusters and conferring them with rich tumor-initiating properties, OVOL can be an important therapeutic target. Reducing OVOL levels could break apart clusters of CTCs in the circulation. These clusters (made of cells with the E/M phenotype) may be the key drivers of metastasis and ‘breaking’ them could deprive them of many advantages of cluster migration—resistance to anoikis, more tumor-initiating potential, ease of extravasation, and finally ‘priming’ for subsequent dissemination [18, 42–44]. Recent diagnostic attempts have mostly focused on isolating individual CTCs [44], however, these results suggest that the most effective diagnostic approach would be to isolate and characterize clusters of CTCs.

Importantly, our study unifies many apparently contradictory experiments such as those indicating that a complete EMT leads to increased stemness [13, 14], MET associates with enhanced stemness [19, 20, 45], and partial EMT phenotype has the maximum stemness [17, 46–48]. We demonstrate that the ‘stemness window’ [46] is quite flexible or tunable on the ‘EMT axis’. Consequently, different phenotypes can gain stemness, either alone or in different combinations among themselves, depending on (a) strengths of ‘feed-forward’ and ‘feed-backward’ coupling between the EMT and stemness circuits, (b) levels of external signals to the circuits such as SNAIL, and (c) ‘phenotypic stability factors’ such as OVOL [49]. Because different cell lines operate in different regimes of coupling strengths and levels of SNAIL and OVOL, each of them is likely to have its own EMT-stemness coupling, slightly different from the other. In an actual tumor, different microenvironments could lead to spatially varying correlations between stemness and motility phenotypes, as recently indicated [15].

Aside from unifying many apparently contradictory studies concerning EMT-stemness interplay, our study also offers new testable predictions. For instance, we predict that the loss or inhibition of OVOL can promote the ability of mesenchymal (M) cells to gain stemness. Loss of OVOL has been shown to enable the transition of hybrid E/M cells to being completely mesenchymal [34], however, the expression of OVOL needs to be measured in mesenchymal stem cells for verifying its predicted role in enabling M cells to be stem-like. Importantly, we present here OVOL as one example of a modulating factor that can fine-tune the E/M state as well as acquisition of stemness. Such a role can also be potentially played by other pro-epithelial genes such as E-cadherin and P-cadherin [50]; the need for additional factors could arise, for instance, because the inhibition of ZEB by OVOL might be relevant in some biological contexts where EMT is operative [34, 35, 41], but not necessarily all. Given the emerging notion that most tumors *in vivo* undergo only a partial EMT [51], it would be important to identify other modulating factors that can fine-tune the hybrid E/M phenotype. The E/M phenotype, sometimes referred to as an ‘EMT-like’ state [48], also needs to be characterized further functionally in the contexts of both physiological and pathological EMT. Here, we characterize the motility phenotypes discretely (E, M and E/M) based on the tristable behavior of the core EMT circuit miR-200/ZEB, however, including other components in the model such as E-cadherin and P-cadherin that are regulated during EMT might fine-tune this characterization to appear more close to being a continuum of phenotypes [52, 53].

Similar to EMT, many other cellular decisions are not necessarily binary but rather ternary and governed by underlying three-way gene circuits. Dynamical characteristics of such three-way gene circuits have been theoretically investigated individually [54–57]. However, to the best of our knowledge, ours is the first computational study that couples two three-way genetic switches to elucidate the operating principles of coupled cellular decision-making. It is not yet clear that how many of our results depend on the specific details of the two circuits considered here. Hence, future studies using more generic models of tristable circuits are required. Further, to investigate the ‘underlying organizing principles' [58] of coupled cellular decision-making in cancer, the decision-making circuits of EMT and stemness should be coupled with circuits regulating other hallmarks of cancer such as deregulated cellular metabolism [58–60].

With an increasing interest in decoding the signaling pathways [36, 61–68] underlying many hallmarks of cancer to elucidate their ‘underlying organizing principles' [58], the theoretical approach presented here can serve as a basis for incorporating other intracellular and extracellular signals, and also aid in investigating the efficacy of different therapeutic strategies that target EMT and/or CSCs.

## MATERIALS AND METHODS

### Model formulation

Our coupled circuit model of EMT and stemness (miR-200/ZEB/LIN28/let-7) has six components - microRNA miR-200 (*μ*_200_), ZEB mRNA (*m_z_*), ZEB protein (*Z*), microRNA let-7 (*μ_l_*), LIN28 mRNA (*m_l_*), and LIN28 protein (*L*)—and includes multiple modes of regulation—transcriptional (ZEB inhibiting miR-200, and self-activation of ZEB), microRNA-mediated (miR-200 inhibiting ZEB and LIN28, and let-7 inhibiting both LIN28 and ZEB), microRNA processing (LIN28′s inhibition of let-7 and self-activation of let-7) and translational (self-activation of LIN28). We generalized and extended the theoretical framework devised by Lu *et al*. [23] for the EMT circuit, and Jolly *et al*. [18] for the stemness circuit, and include (a) two coupling links, ‘feed-forward coupling’ (inhibition of LIN28 by miR-200), and ‘feed-backward coupling’ (inhibition of ZEB by let-7), and (b) coupling of miR-200/ZEB with OVOL. Except for miRNA-mediated regulation with a large number of binding sites of miRNA on its target mRNA (such as for miR-200 inhibiting ZEB with 6 binding sites), all other effects have been modeled via shifted Hill functions (*H^S+^* (X, λ) for activation and *H^S−^* (X, λ) for inhibition). Shifted Hill functions are defined as the weighted sum of positive Hill function H^+^(X) and negative Hill function H^−^(X) − *H^S^*(*X, λ_X, Y_*) = *H*^−^ (*X*) + λ*H*^+^ (*X*), where λ_*X, Y*_ represents the fold-change in the production rate of Y due to regulation by X. For activation, λ > 1; for inhibition, λ < 1; and for no effect, λ = 1 [23]. For the two links coupling the circuits (miR-200 inhibiting LIN28 and let-7 inhibiting ZEB), the strength of inhibition is denoted by α = 1- λ, 0 < α < 1; larger the value of α, stronger the inhibition, and thus shifted Hill function is denoted by *H^S−^* (*X, α_X, Y_*) = *H^−^* (*X*) + λ*H*^+^ (*X*) = 1 − *αH*^+^ (*X*). Effects of miR-200 on ZEB include both degradation of mRNA and translational inhibition by miRNAs that can themselves be degraded after binding and forming complexes with the mRNAs [23].

SNAIL and NF-kB are treated as external signals for EMT and stemness circuits respectively with their effects captured by shifted Hill functions. Similarly, including OVOL in our analysis entails including two more components: OVOL mRNA (*m_O_*) and OVOL protein (*O*). The regulations involving OVOL and miR-200/ZEB loop as well as the external activation or inhibition signal on OVOL is also captured by shifted Hill functions. Details of the model construction for the miR-200/ZEB/LIN28/let-7/OVOL and parameter values used in the model can be found in SI sections 1, 2, and 3 and Tables S1-5. The model is quite robust with respect to changes in parameter values as discussed in SI section 4 (Figure S1). Codes were implemented in Python using PyDSTool [69]. Figures 2 and S2 were drawn in MATLAB.

## SUPPLEMENTARY INFORMATION FIGURES AND TABLES


