# Supplementary material for: Coupling the modules of EMT and stemness: A tunable ‘stemness window’ model
Source: Oncotarget. 2015 Jul 23;6(28):25161–74. doi: 10.18632/oncotarget.4629 (PMC4694822; doi:10.18632/oncotarget.4629)
Supplement: Supplementary file 1 [file oncotarget-06-25161-s001.pdf]

SUPPLEMENTARY INFORMATION

REGULATORY CIRCUIT DETAILS

EMT regulatory network

EMT regulatory network includes two highly interconnected modules – miR-34/SNAIL and miR-200/ZEB, both of which are mutual inhibitory circuits[1–3]. miR-34/SNAIL has been shown to act as a noise-buffering integrator, while miR-200/ZEB acts as a three-way switch allowing the co-existence of three phenotypes – epithelial (E - high miR-200, low ZEB), hybrid epithelial/ mesenchymal (E/M - intermediate miR-200, intermediate ZEB), and mesenchymal (M - low miR-200, high ZEB), and acts as the decision-making module during EMT. Coupling miR-34/SNAIL to miR-200/ZEB does not change the qualitative behavior of EMT decision-making [4, 5]; hence we treat SNAIL as the external input signal to the miR-200/ZEB circuit.

The miR-200 family contains two subgroups based on the seed sequences – one subgroup comprises miR-141 and miR-200a, and the other includes miR-200b, miR-200c, and miR-429. The transcription factor ZEB family includes two isoforms – ZEB1 and ZEB2. The 3′ UTR region of ZEB1 has a total of eight conserved binding sites for miR-200 - three for the first subgroup and five for the second subgroup. The 3′ UTR region of ZEB2 has nine conserved binding sites for miR-200, specifically three for the first subgroup and six for the second subgroup [6]. Experimental evidence suggests that the expression of miR-200c standalone can induce MET and restore the expression of E-cadherin [7]. Therefore, here, in our miR-200/ZEB module, we considered six binding sites on the 3′ UTR region of ZEB for the binding of miR-200 and three binding sites on the promoter region of miR-200 for the binding of ZEB. In addition, ZEB can also activate its own transcription through SMAD complexes [8] and we assume two binding sites in the promoter region of ZEB for its self-activation. SNAIL is a well-known EMT inducer, which can transcriptionally inhibit miR-200 and activate ZEB [2, 9]. Both these regulations have been assumed to happen through two binding sites [4].

Stemness regulatory network

Stemness is governed by a mutually inhibitory loop between RNA-binding factor family LIN28 and microRNA family let-7. Being RNA-binding factors, LIN28A and LIN28B, the two members of LIN28 family, can repress the biogenesis of let-7 by blocking its processing to mature miRNAs. LIN28B inhibits the processing of primary let-7 transcripts by the microprocessor Drosha, and LIN28A recruits a TUTase (Zcchc11/ TUT4) to block the processing of let-7 precursors by Dicer in the cytoplasm [10, 11]. Being a microRNA, let-7 can inhibit the translation process of LIN28 [10]. Further, let-7 can promote its own processing and LIN28 can promote its own translation by binding to seven consensus sites on its own mRNA [12, 13].

LIN28/let-7 circuit also behaves as a three-way switch – with the three states being U (Up – high LIN28, low let-7), D (Down – low LIN28, high let-7) and D/U (Down/Up – medium LIN28, medium let-7) [14]. LIN28 promotes the translation of pluripotency factor OCT4 [15]. Both very high and very low levels of OCT4 lead to differentiation, but its intermediate levels are strongly associated with gaining stemness [16–19]. Therefore, cells with intermediate levels of LIN28 (or D/U state) are most likely to lie in the 'stemness window' or correspond with the maximum likelihood of gaining stemness.

Coupling of the two core networks

These two core networks are coupled in two ways: miR-200 inhibits LIN28 by binding to its mRNA, and let-7 inhibits HMGA2 that activates ZEB, hence indirectly repressing ZEB [20–22].

MODEL FORMULATION

In the combined circuit of miR-200/ZEB/LIN28/let-7, there are multiple modes of regulation – transcriptional, translational, miRNA-mediated regulation, and regulation of miRNA processing. For all modes except miRNA-mediated regulation with a large number of binding sites of miRNA on its target mRNA, we use shifted Hill functions $H^S(X, \lambda)$ defined as $H^S(X, \lambda) = H^-(X) + \lambda H^+(X)$, a weighted sum of positive and negative Hill functions. Parameter λ in the shifted Hill functions is the weight factor that represents fold-change in production rate from its basal level, due to the binding of regulatory factor. For activation, $\lambda > 1$ and shifted Hill function is represented by H^{S+}; for repression, $0 < \lambda < 1$ and shifted Hill function is represented by H^{S-}; and for no change, $\lambda = 1$. $\lambda_{X,Y}$ denotes the effect of X on Y. Transcriptional regulation has been canonically modeled using Hill function [23], and inhibition of let-7 by LIN28 has been shown experimentally to behave like an inhibitory Hill function [24].

The terms representing miRNA-mediated translational effects capture both actions of miRNA - mRNA degradation and mRNA sequestration so as to prevent translation. Since a microRNA is usually 22nt long, and the length of the seed sequence it recognizes on a mRNA is only 7~8 nt, here we assume that the bindings of different microRNAs are independent. We assume the binding rate between microRNA and mRNA is r_+ and

the unbinding rate is r_-, and that these binding/unbinding processes between microRNA and mRNA are much faster as compared to production and degradation of proteins, therefore miRNA and mRNA are always in equilibrium, i.e. $r_+\mu[m_i] = r_-[m_{i+1}]$, where μ represents the concentration of microRNA, $[m_i]$ represents the mRNA bound with i microRNAs and $[m_{i+1}]$ represents the mRNA bound with (i+1) microRNAs. Therefore, $[m_i] = (\mu/\mu_0)^i[m_0]$, where $\mu_0 = r_-/r_+$. All terms $[m_i]$ should satisfy $\sum_{i=0}^{n} C_n^i[m_i] = m$, where C_n^i is the number of combinations for i terms in n positions, which is defined as $C_n^i = n!/(i!(n-i)!)$ and m is total concentration of the mRNA. Since there are C_n^i different possibilities for i number of microRNA (miR) molecules binding to mRNA with n binding sites for that particular microRNA (miR), therefore $[m_i] = mM_n^i(\mu)$, where $M_n^i(\mu) = (\mu/\mu_0)^i/(1+\mu/\mu_0)^n$. Thus, the translation rate is given by $mL(\mu) = \sum_{i=0}^{n} l_i C_n^i[m_i] = m\sum_{i=0}^{n} l_i C_n^i M_n^i(\mu)$, where l_i is the individual translation rate of an mRNA when bound to i microRNAs. The term describing the degradation of the mRNA due to binding with the microRNA is $mY_m(\mu) = \sum_{i=0}^{n} \gamma_{mi} C_n^i[m_i] = m\sum_{i=0}^{n} \gamma_{mi} C_n^i M_n^i(\mu)$, where γ_{mi} is the individual degradation rate of mRNA bound to i molecules of miRNA. Similarly, the term denoting the degradation rate of microRNA due to binding to many mRNAs is $mY_\mu(\mu) = \sum_{i=0}^{n} i\gamma_{\mu i} C_n^i[m_i] = m\sum_{i=0}^{n} i\gamma_{\mu i} C_n^i M_n^i(\mu)$, where $\gamma_{\mu i}$ is the individual degradation rate for a microRNA molecule when i molecules of microRNA are bound to a mRNA. For small number of binding sites (~2) of microRNA on its target mRNA, the net silencing effects of microRNA can be approximated as a Hill function [4]. Therefore, for the cases when let-7 inhibits LIN28 and ZEB, and when miR-200 inhibits LIN28, shifted Hill functions are used to represent the effects of microRNAs.

Specifically in the two links that represent couple EMT and stemness core circuits – miR-200 inhibiting LIN28 and let-7 inhibiting ZEB – the strength of the inhibition is defined as $\alpha = 1 - \lambda$, $0 < \alpha < 1$, therefore the larger value of α represents stronger inhibition. Shifted Hill function formula for these two inhibitions can thus be denoted by $H^{S-}(B, \alpha) = H^-(B) + \lambda H^+(B) = 1 - \alpha H^+(B)$, where $0 < \alpha < 1$.

The coupled circuits of EMT and stemness (miR-200/ZEB/let-7/LIN28) - contain six components - microRNA miR-200 (μ_{200}), ZEB mRNA (m_Z), ZEB protein (Z), microRNA let-7 (μ_l), LIN28 mRNA (m_l), and LIN28 protein (L). On including OVOL, two more components – OVOL mRNA($m_{\_O}$) and OVOL protein (O) are also incorporated.

The dynamics of miR-200 (μ_{200}) can be described by the following equation:

$$\frac{d\mu_{200}}{dt} = g_{\mu_{200}} H^{S-}(Z, \lambda_{Z,\mu_{200}}) H^{S-}(S, \lambda_{S,\mu_{200}}) - m_Z Y_\mu(\mu_{200}) - k_{\mu_{200}}\mu_{200} \quad (1)$$

where $g_{\mu_{200}}$ and $k_{\mu_{200}}$ are innate production and degradation rates of miR-200 respectively. $H^{S-}(Z, \lambda_{Z,\mu_{200}})$ represents transcriptional inhibition of miR-200 by ZEB and $H^{S-}(S, \lambda_{S,\mu_{200}})$ represents transcriptional inhibition of miR-200 by SNAIL. $Y_\mu(\mu_{200})$ represents the degradation rate of miR-200 due to forming a complex with ZEB mRNAs.

The dynamics of ZEB mRNA (m_Z) and ZEB protein (Z) are described by the following equations:

$$\frac{dm_Z}{dt} = g_{m_Z} H^{S+}(Z, \lambda_{Z,m_Z}) H^{S+}(S, \lambda_{S,m_Z}) - m_Z Y_m(\mu_{200}) - k_{m_Z} m_Z \quad (2)$$

$$\frac{dZ}{dt} = g_Z m_Z L(\mu_{200}) H^{S-}(u_l, \alpha_{u_l,m_Z}) - k_Z Z \quad (3)$$

where g_{m_Z} and g_Z are the innate production rates of ZEB mRNA and ZEB protein respectively, and k_{m_Z} and k_Z are their respective innate degradation rates. $H^{S+}(Z, \lambda_{Z,m_Z})$ denotes transcriptional self-activation of ZEB, and $H^{S+}(S, \lambda_{S,m_Z})$ denotes transcriptional activation of ZEB by SNAIL. $Y_m(\mu_{200})$ represents the degradation of ZEB mRNA due to forming mRNA-miRNA complexes with miR-200, $L(\mu_{200})$ denotes the translational inhibition of ZEB by miR-200, and $H^{S-}(u_l, \alpha_{u_l,m_Z})$ represents the inhibition from let-7.

When including OVOL, equation (1) for dynamics of miR-200 and equation (2) for ZEB mRNA are changed accordingly as follows:

$$\frac{d\mu_{200}}{dt} = g_{\mu_{200}} H^{S-}(O, \lambda_{O,\mu_{200}}) H^{S-}(Z, \lambda_{Z,\mu_{200}}) H^{S-}(S, \lambda_{S,\mu_{200}}) - m_Z Y_\mu(\mu_{200}) - k_{\mu_{200}}\mu_{200} \quad (4)$$

$$\frac{dm_Z}{dt} = g_{m_Z} H^{S-}(O, \lambda_{O,m_z}) H^{S+}(Z, \lambda_{Z,m_Z}) H^{S+}(S, \lambda_{S,m_Z}) - m_Z Y_m(\mu_{200}) - k_{m_Z} m_Z \quad (5)$$

where $H^{S-}(O, \lambda_{O,\mu_{200}})$ represents inhibition of miR-200 by OVOL and $H^{S-}(O, \lambda_{O,m_Z})$ represents the inhibition of ZEB by OVOL.

Dynamics of OVOL mRNA (m_O) and OVOL protein (O) are described by these equations:

$$\frac{dm_O}{dt} = g_{m_O} H^{S-}(O, \lambda_{O,m_O}) H^{S-}(Z, \lambda_{Z,m_O}) - k_{m_O} m_O \quad (6)$$

$$\frac{dO}{dt} = g_O m_O - k_O O \tag{7}$$

where g_{m_O} and g_O are innate production rates of OVOL mRNA and protein respectively, and k_{m_O} and k_O are their respective degradation rates. $H^{S-}(O, \lambda_{O,m_O})$ represents the transcriptional self-inhibition of OVOL, and $H^{S-}(Z, \lambda_{Z,m_O})$ represents the transcriptional inhibition of OVOL by ZEB.

The dynamics of let-7 (μ_l) is described by the following equation:

$$\frac{d\mu_l}{dt} = g_{\mu_l} H^{S+}(\mu_l, \lambda_{\mu_l,\mu_l}) H^{S-}(L, \lambda_{L,\mu_l}) H^{S+}(N, \lambda_{N,\mu_l}) - k_{\mu_l}\mu_l \tag{8}$$

where g_{μ_l} and k_{μ_l} are the innate production and degradation rates of let-7 respectively, $H^{S+}(\mu_l, \lambda_{\mu_l,\mu_l})$ represents the self-activation of let-7, $H^{S-}(L, \lambda_{L,\mu_l})$ represents inhibition by protein LIN28 and $H^{S+}(N, \lambda_{N,\mu_l})$ denotes activation by external signal (protein NF-kB (represented by N)).

Dynamics of LIN28 mRNA (m_l) and protein (L) are described by the following equations:

$$\frac{dm_l}{dt} = g_{m_l} - k_{m_l} m_l \tag{9}$$

$$\frac{dL}{dt} = g_L m_l H^{S-}(\mu_{200}, \alpha_{u_{200},L}) H^{S+}(L, \lambda_{L,L}) H^{S+}(N, \lambda_{N,L}) H^{S-}(l, \lambda_{l,L}) - k_L L \tag{10}$$

where g_{m_l} and g_L are innate production rates of LIN28 mRNA and protein respectively, and k_{m_l} and k_L are their respective degradation rates. $H^{S+}(L, \lambda_{L,L})$ denotes translational self-activation of LIN28, $H^{S+}(N, \lambda_{N,L})$ represents transcriptional activation of LIN28 by external signal (NF-kB (denoted by N)), $H^{S-}(\mu_{200}, \alpha_{u_{200},L})$ denotes inhibition of LIN28 by miR-200, and $H^{S-}(l, \lambda_{l,L})$ denotes translational inhibition of LIN28 by let-7.

PARAMETERS ESTIMATION

The innate degradation rates of miRNAs, mRNAs, and proteins are selected based on their experimentally measured half-lives. Typical half-life of mammalian proteins is around 10 hours [25], so we chose 0.1/hour as the degradation rate for protein ZEB, OVOL and LIN28. The half-life of mRNA is around several hours [26], so we chose 0.5/ hour as the degradation rate for the mRNAs of ZEB, OVOL and LIN28. Generally, microRNA is more stable than mRNA [27, 28], therefore we chose 0.05/hour as the degradation rate for miR-200 and let-7.

The expression levels of various molecules were estimated based on the typical concentrations and ratios in eukaryotic cells. The typical length scale for dimensions of a mammalian cell is around $10\mu m$ and typical protein concentration is in the range $10\ nM \sim 1\ \mu M$ [29]. Hence, there are around $6 \times 10^{23} \times 10^{-6}/10^{-3} \times (10 \times 10^{-6})^3$ or about one million molecules. Because the typical ratio of protein/mRNA is around 2800 [30], the level of mRNA should be in range of thousands of molecules. In addition, the number of microRNA is around ten thousand molecules [31]. Based on these typical levels, we chose production rates accordingly. The typical translation rate for one gene is around 140 proteins/(mRNA*hour) [30], here we chose 100 proteins/(mRNA*hour) for ZEB and 200 proteins/(mRNA*hour) for LIN28. The coupling strength parameters - $\alpha 1$ and $\alpha 2$ - vary from 0 (no coupling) to 1 (very strong coupling). All parameters used in the model have been detailed in Tables S1–S5.

PARAMETERS SENSITIVITY ANALYSIS

To test the sensitivity of our predictions based on the parameters we listed above, we conduct parameter sensitivity analysis by varying each parameter at one time. All the parameters – production rates, degradation rates, thresholds and weight factors in the shifted Hill functions are varied by ± 20%. The number of binding sites for the different interactions have been kept fixed because most of them are directly determined from experimental data.

First, we conduct parameter sensitivity for EMT regulatory circuit – miR-200/ZEB. For some values of the driving signal SNAIL, this circuit typically has three states - epithelial (E), hybrid epithelial/mesenchymal (E/M), and mesenchymal (M). Here, we measure how changes in the parameters affect the range of SNAIL levels for which the hybrid E/M phenotype exists (either alone in or combination with other phenotypes). Not surprisingly, the absolute values of SNAIL levels for the existence of E/M phenotype changes with every parameter change (Figure S1), but here we focus on the range of SNAIL levels (i.e. the difference in the minimum and the maximum values of SNAIL) for which the cells can attain any of the three phenotypes, and not the absolute levels of SNAIL. Comparing with the control case (case 0, denoting the parameters listed above in the Tables S1–S5), the changes in the production rates and degradation rates of all species don't affect the range of SNAIL levels for which the hybrid state exists very much, as shown in Figure S1A. However, the range of SNAIL levels for which the hybrid E/M state exists decreases when threshold of the shifted Hill function representing the inhibition by ZEB on miR-200 decreases (case G2), threshold of the shifted Hill function representing the self-activation of ZEB increases (case H1), the weight factor in the

shifted Hill function representing the self-activation of ZEB increases (case N1) and weight factor in the shifted Hill function representing the activation on ZEB by SNAIL increases (case O1). All these cases represent an increase in effective ZEB levels that can inhibit miR-200 and hence drive EMT more strongly. In contrast, the range of SNAIL levels for which the hybrid E/M state exists increases when the threshold in the shifted Hill function representing the inhibition of miR-200 by ZEB increases (case G1), the threshold in the shifted Hill function representing ZEB self-activation decreases (case H2), the weight factor in the shifted Hill function representing ZEB self-activation decreases (case N2) and the weight factor in the shifted Hill function representing the activation of ZEB by SNAIL decreases (case O2). During all these cases, the effective ZEB levels that can inhibit miR-200 decreases, which result in a higher effective miR-200 levels and contribute to a larger range for which E/M exists (Figure S1B). These results suggest that changes in the parameters which decrease the levels of miR-200 decrease the overall range of SNAIL levels for existence of E/M phenotype, or in other words, the model is sensitive towards low values of miR-200.

Similar sensitivity analysis was conducted for stemness regulatory circuit – LIN28/ let-7 in our previous manuscript [14], suggesting that changes in parameters which cause a decrease in the levels of let-7 result in the decrease of range of NF-kB levels for which the intermediate D/U state (medium LIN28, medium let-7) exists.

As the final step, we also varied the 'stemness window' parameters that are based on experimental data suggesting that the intermediate levels of OCT4 correspond to the maximum likelihood of being 'stem-like', and that both strong overexpression and strong down-regulation can force the cell to differentiate [16–18]. Such intermediate OCT4 levels are usually attained by intermediate levels of LIN28, i.e. the (D/U) state [14]. OCT4 is an output of the coupled circuit, and translational activation of OCT4 by LIN28 is represented by a positive Hill function $H^{+} = (L_I/L_{I_0}^{O_c})^2/(1 + (L_I/L_{I_0}^{O_c})^2)$, where L_I represents LIN28 levels, and $L_{I_0}^{O_c}$ represents threshold levels of LIN28 for OCT4 activation = 480 * 10^3 molecules. The value of this Hill function lies between 0 and 1. Here, based on experimental data, we consider a 'guessed range' for the levels of OCT4 pertaining to 'stemness window' on this scale of (0–1), i.e. (0.25–0.65). If OCT4 values pertaining to any phenotype lies in this regime, that phenotype is within the 'stemness window' or has a heightened likelihood of gaining stemness. The LIN28 threshold levels (= 480 * 10^3 molecules) are chosen based on the overall scale of LIN28 values (~1000 * 10^3 molecules). Thus, the range of corresponding LIN28 levels for the 'stemness window' is [277 * 10^3, 655 * 10^3] molecules. This range was varied by 20% to understand how the definition of the 'stemness window' affects our results, and we did not find any significant change in the phase diagrams obtained, for instance, results pertaining to inhibiting OVOL as shown in Figure 6, thereby suggesting the robustness of the model to our definition of 'stemness window'.

Collectively, the parameter sensitivity analysis suggests that as long as the levels of let-7 or miR-200 are not too low, our model is quite robust to parameter changes. This observation highlights an important facet – extremely low levels of let-7 and/or miR-200 will anyway render one or both coupling links irrelevant (Figure S2) – inhibition from miR-200 to LIN28 and/or inhibition from let-7 to ZEB would be more or less independent of α1 and/or α2 respectively. Therefore, in the parameter regime where coupling links can potentially largely affect the dynamics of the two circuits, our model is quite robust.

STABLE STATES OF THE COUPLED EMT AND STEMNESS CIRCUITS AT DIFFERENT SNAIL LEVELS

Inhibition of miR-200 on LIN28 and inhibition of let-7 on ZEB couple the two circuits – miR-200/ZEB and LIN28/let-7 together. We explore how different initial conditions, i.e. different levels of SNAIL at no coupling affect the stable steady states obtained at a given set of coupling parameters for the combined circuit (Figure 3–5).

Starting from monostable phase {E} as enabled by SNAIL levels= 180 * 10^3 molecules, α1 (strength of inhibition of LIN28 by miR-200) affects the coupled dynamics much more than α2 (strength of inhibition of ZEB by let-7) because levels of miR-200 are quite high initially and hence any changes in the set of stable states change depends much more on α1 than α2 (Figure S2B). Conversely, starting from monostable phase {M} as enabled by SNAIL = 240 * 10^3 molecules, α1 (strength of inhibition of LIN28 by miR-200) affects the coupled dynamics of EMT and stemness much less than α2 (strength of inhibition of ZEB by let-7) because levels of miR-200 are quite low initially and therefore the set of stable states change depends much more largely on α2, specially at lower values of α1 (Figure S2A).

STABLE STEADY STATES OF THE COUPLED CIRCUITS AT DIFFERENT VALUES OF (α1, α2)

In Figure 2, we illustrate that two phases that have the same number of stable states but are obtained

at different ranges of coupling parameters (α1, α2) have the different set of stable states. Here, we show more examples denoting the same principle (Figure S3).

OVOL PULLS THE DYNAMIC 'STEMNESS WINDOW' TOWARDS E END OF EMT AXIS

In Figure 4, we analyze how OVOL affects the likelihood of the phenotypes E, M and E/M in gaining stemness, when cells are in monostable {M} phase enabled by the SNAIL levels = 340 * 10^3 molecules at no coupling between the circuits (α1 = α2 = 0). Here, we show the case when SNAIL levels = 360 * 10^3 molecules at no coupling (α1 = α2 = 0). Under very strong coupling, in the absence of OVOL, only the mesenchymal (M) phenotype can gain stemness (Figure S4A), but in presence of OVOL, only hybrid (E/M) phenotype can gain stemness under very strong coupling (Figure S4B). These results resonate with those presented in the main text that OVOL can aid the hybrid E/M phenotype to gain stemness and preclude M phenotype from gaining stemness.

We further explore the effect of OVOL in mediating the positioning of the 'stemness window' on the 'EMT axis'. Coupling OVOL to miR-200/ZEB enables a special feature not seen in the miR-200/ZEB circuit without OVOL – a monostable {E/M} phase [32]. Starting from this phase initially as enabled by SNAIL levels = 300 * 10^3 molecules, we add an external activation signal on OVOL to explore how the effect of over-expression of OVOL on EMT-stemness interplay. Overexpressing OVOL enables the E phenotype to gain stemness for almost the entire range of (α1, α2). Further, the hybrid E/M phenotype can also gain stemness at low α2 (weak inhibition of ZEB by let-7) (Figure S5). This result is in stark contrast to the phase diagram obtained when OVOL was inhibited by an external signal (Figure 6) where for low α2 (weak inhibition of ZEB by let-7), both E/M and M phenotypes can gain stemness, as well as the epithelial phenotype cannot gain stemness unless accompanied by the E/M phenotype for any values of (α1, α2).

Overall, OVOL can pull the 'stemness window' towards E end of the axis, and are consistent with previous experimental as well as theoretical observations [32, 33] that the overexpression of OVOL induces a Mesenchymal to Epithelial Transition (MET), thereby enabling a much higher likelihood of epithelial phenotype to gain stemness. However, in the context of cancer, only rarely can cell lines be classified as purely epithelial; they are usually EMT-prone due to factors such as genomic plasticity, hence, OVOL is down-regulated during tumor progression as shown earlier [33]. Consequently, E phenotype might not be likely to gain stemness in the context of cancer (type III EMT) as in the case of wound healing and embryonic development (types I and II EMT).

REFERENCES

1. Bracken CP, Gregory PA, Kolesnikoff N, Bert AG, Wang J, Shannon MF, Goodall GJ. A double-negative feedback loop between ZEB1-SIP1 and the microRNA-200 family regulates epithelial-mesenchymal transition. Cancer Res. 2008; 68:7846–54.
2. Burk U, Schubert J, Wellner U, Schmalhofer O, Vincan E, Spaderna S, Brabletz T. A reciprocal repression between ZEB1 and members of the miR-200 family promotes EMT and invasion in cancer cells. EMBO Rep. 2008; 9:582–9.
3. Siemens H, Jackstadt R, Hünten S, Kaller M, Menssen A, Götz U, Hermeking H. miR-34 and SNAIL form a double-negative feedback loop to regulate epithelial-mesenchymal transitions. Cell Cycle. 2011; p.4256–71.
4. Lu M, Jolly MK, Levine H, Onuchic JN, Ben-Jacob E. MicroRNA-based regulation of epithelial-hybrid-mesenchymal fate determination. Proc Natl Acad Sci U S A. 2013; 110:18174–9.
5. Lu M, Jolly MK, Onuchic J, Ben-Jacob E. Toward Decoding the Principles of Cancer Metastasis Circuits. Cancer Res. 2014; 74:4574–87.
6. Brabletz S, Brabletz T. The ZEB/miR-200 feedback loop—a motor of cellular plasticity in development and cancer? EMBO Rep. 2010; 11:670–7.
7. Hurteau GJ, Carlson JA, Roos E, Brock GJ. Stable expression of miR-200c alone is sufficient to regulate TCF8 (ZEB1) and restore E-cadherin expression. Cell Cycle. 2009; 8:2064–9.
8. Hill L, Browne G, Tulchinsky E. ZEB/miR-200 feedback loop: at the crossroads of signal transduction in cancer. Int J Cancer. [Internet] 2013; Feb 15. [cited 2013 Nov 17]; 132:745–54. Available from: http://www.ncbi.nlm.nih.gov/pubmed/22753312.
9. Guaita S, Puig I, Franci C, Garrido M, Dominguez D, Batlle E, Sancho E, Dedhar S, De Herreros AG, Baulida J. Snail induction of epithelial to mesenchymal transition in tumor cells is accompanied by MUC1 repression and ZEB1 expression. J Biol Chem. 2002; 277:39209–16.
10. Yang X, Lin X, Zhong X, Kaur S, Li N, Liang S, Lassus H, Wang L, Katsaros D, Montone K, Zhao X, Zhang Y, Bützow R, et al. Double-negative feedback loop between reprogramming factor LIN28 and microRNA let-7 regulates aldehyde dehydrogenase 1-positive cancer stem cells. Cancer Res. 2010; 70:9463–72.
11. Piskounova E, Polytarchou C, Thornton JE, Lapierre RJ, Pothoulakis C, Hagan JP, Iliopoulos D, Gregory RI. Lin28A and Lin28B inhibit let-7 MicroRNA biogenesis by distinct mechanisms. Cell. 2011; 147:1066–79.

12. Hafner M, Max KE a, Bandaru P, Morozov P, Gerstberger S, Brown M, Molina H, Tuschl T. Identification of mRNAs bound and regulated by human LIN28 proteins and molecular requirements for RNA recognition. RNA. 2013; 19:613–26.
13. Zisoulis DG, Chang RK, Kai ZS, Pasquinelli AE. Autoregulation of microRNA biogenesis by let-7 and Argonaute. Nature. 2012; p.541–4.
14. Jolly MK, Huang B, Lu M, Mani SA, Levine H, Ben-Jacob E. Towards elucidating the connection between epithelial − mesenchymal transitions and stemness. J R Soc Interface. 2014; 11:20140962.
15. Qiu C, Ma Y, Wang J, Peng S, Huang Y. Lin28-mediated post-transcriptional regulation of Oct4 expression in human embryonic stem cells. Nucleic Acids Res. 2010; 38:1240–8.
16. Niwa H, Miyazaki J, Smith AG. Quantitative expression of Oct-3/4 defines differentiation, dedifferentiation or self-renewal of ES cells. Nat Genet. 2000; 24:372–6.
17. Theunissen TW, Van Oosten AL, Castelo-Branco G, Hall J, Smith A, Silva JCR. Nanog overcomes reprogramming barriers and induces pluripotency in minimal conditions. Curr Biol. 2011; 21:65–71.
18. Karwacki-Neisius V, Göke J, Osorno R, Halbritter F, Ng JH, Weiße AY, Wong FCK, Gagliardi A, Mullin NP, Festuccia N, Colby D, Tomlinson SR, Ng HH, et al. Reduced Oct4 expression directs a robust pluripotent state with distinct signaling activity and increased enhancer occupancy by Oct4 and Nanog. Cell Stem Cell. 2013; 12:531–45.
19. Shu J, Wu C, Wu Y, Li Z, Shao S, Zhao W, Tang X, Yang H, Shen L, Zuo X, Yang W, Shi Y, Chi X, et al. Induction of pluripotency in mouse somatic cells with lineage specifiers. Cell [Internet]. Elsevier Inc. 2013; 153:963–75. Available from: http://dx.doi.org/10.1016/j.cell.2013.05.001.
20. Kong D, Banerjee S, Ahmad A, Li Y, Wang Z, Sethi S, Sarkar FH. Epithelial to mesenchymal transition is mechanistically linked with stem cell signatures in prostate cancer cells. PLoS One. 2010; Jan 5:e12445.
21. Yong SL, Dutta A. The tumor suppressor microRNA let-7 represses the HMGA2 oncogene. Genes Dev. 2007; 21:1025–30.
22. Guo L, Chen C, Shi M, Wang F, Chen X, Diao D, Hu M, Yu M, Qian L, Guo N. Stat3-coordinated Lin-28-let-7-HMGA2 and miR-200-ZEB1 circuits initiate and maintain oncostatin M-driven epithelial-mesenchymal transition. Oncogene. 2013; 32:5272–82.
23. Macía J, Widder S, Solé R. Why are cellular switches Boolean? General conditions for multistable genetic circuits. J Theor Biol. 2009; 261:126–35.
24. Desjardins A, Yang A, Bouvette J, Omichinski JG, Legault P. Importance of the NCp7-like domain in the recognition of pre-let-7g by the pluripotency factor Lin28. Nucleic Acids Res. 2012; 40:1767–77.
25. Eden E, Geva-Zatorsky N, Issaeva I, Cohen A, Dekel E, Danon T, Cohen L, Mayo A, Alon U. Proteome Half-Life Dynamics in Living Human Cells. Science (80-). 2011; Feb 331:764–8.
26. Yang E, van Nimwegen E, Zavolan M, Rajewsky N, Schroeder M, Magnasco M, Darnell JE. Decay rates of human mRNAs: correlation with functional characteristics and sequence attributes. Genome Res. 2003; Aug 13: 1863–72.
27. Khanin R, Vinciotti V. Computational modeling of post-transcriptional gene regulation by microRNAs. J Comput Biol A J Comput Mol Cell Biol. 2008; Apr 15:305–16.
28. Gantier MP, McCoy CE, Rusinova I, Saulep D, Wang D, Xu D, Irving AT, Behlke MA, Hertzog PJ, Mackay F, Williams BRG. Analysis of microRNA turnover in mammalian cells following Dicer1 ablation. Nucleic Acids Res. 2011; Jul 39:5692–703.
29. Milo R, Jorgensen P, Moran U, Weber G, Springer M. BioNumbers—the database of key numbers in molecular and cell biology. Nucleic Acids Res. 2010; Jan 38:D750–3.
30. Schwanhäusser B, Busse D, Li N, Dittmar G, Schuchhardt J, Wolf J, Chen W, Selbach M. Global quantification of mammalian gene expression control. Nature. 2011; May 473:337–42.
31. Lim LP, Lau NC, Weinstein EG, Abdelhakim A, Yekta S, Rhoades MW, Burge CB, Bartel DP. The microRNAs of Caenorhabditis elegans. Genes Dev. 2003; Apr 17: 991–1008.
32. Jia D, Jolly MK, Boareto M, Parsana P, Mooney SM, Pienta KJ, Levine H, Ben-Jacob E. OVOL guides the epithelial-hybrid-mesenchymal transition. Oncotarget. 2015; 17:15436-48 .
33. Roca H, Hernandez J, Weidner S, McEachin RC, Fuller D, Sud S, Schumann T, Wilkinson JE, Zaslavsky A, Li H, Maher CA, Daignault-Newton S, Healy PN, et al. Transcription Factors OVOL1 and OVOL2 Induce the Mesenchymal to Epithelial Transition in Human Cancer. PLoS One. 2013; 8:e76773.

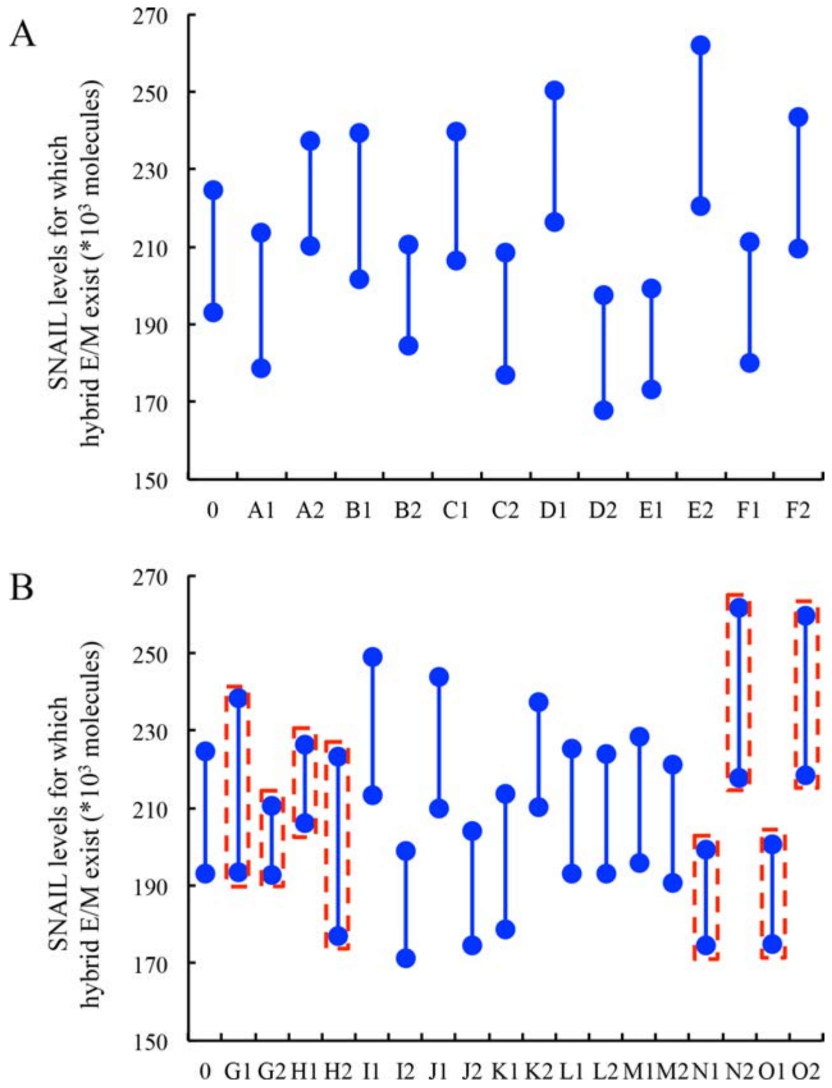

Supplementary Figure S1: Parameter sensitivity analysis for the EMT regulatory circuit miR-200/ZEB driven by input signal SNAIL. Alphanumeric codes on x-axis represent the cases with different changed parameters by ± 20%. Number 1 after the alphanumeric code represents the increase of the parameter by 20% and number 2 after the alphanumeric code represents the decrease of the parameter by 20%. Case 0 represents the result in this work, which is the control case. **A.** represents the cases with ± 20% changes in the production and degradation rates. A1 and A2 represent the increase and decrease in the degradation rate of miR-200 (denoted by $k_{\mu_{200}}$) by 20% respectively. B1 and B2 represent the increase and decrease in the degradation rate of ZEB mRNA by 20% (denoted by k_{m_z}) respectively. C1 and C2 represent the increase and decrease in the degradation rate of ZEB protein (denoted by k_z) by 20% respectively. D1 and D2 represent respectively the increase and decrease in the production rate of miR-200 (denoted by $g_{\mu_{200}}$) by 20%. E1 and E2 represent the increase and decrease in production rate of ZEB mRNA (denoted by g_{m_z}) by 20% respectively. F1 and F2 represent the increase and decrease in the production rate of ZEB protein (g_Z) by 20% respectively. **B.** represents the cases with ± 20% changes in the thresholds and weight factors in the shifted Hill functions. G1 and G2 represent respectively the increase and decrease in the threshold levels for the inhibition of miR-200 by ZEB (denoted by $Z^0_{\mu_{200}}$) by 20%. H1 and H2 represent respectively the increase and decrease in threshold levels of the shifted Hill function representing ZEB self-activation (denoted by $Z^0_{m_Z}$) by 20%. I1 and I2 represent the 20% increase and decrease in the threshold levels of SNAIL inhibition on miR-200 (denoted by $S^0_{\mu_{200}}$) respectively. J1 and J2 represent the increase and decrease in the threshold levels of SNAIL activation on ZEB (denoted by $S^0_{m_Z}$) by 20%. K1 and K2 represent the increase and decrease in the threshold levels of miR-200 (denoted by μ^0_{200}) by 20%. L1 and L2 represent the increase and decrease in the weight factor of ZEB inhibition on miR-200 (denoted by $\lambda_{Z,\mu_{200}}$) by 20%. M1 and M2 represent the respective increase and decrease in the weight factor of SNAIL inhibition on miR-200 (denoted by $\lambda_{S,\mu_{200}}$) by 20%. N1 and N2 represent the respective increase and decrease in the weight factor of ZEB self-activation (denoted by λ_{Z,m_Z}) by 20%. O1 and O2 denote respective increase and decrease in weight factor of SNAIL activation on ZEB (denoted by λ_{S,m_Z}) by 20%. Dotted rectangles highlight the cases when the range of SNAIL values for which the cells can attain E/M phenotype vary the most, as compared to the control (case 0).

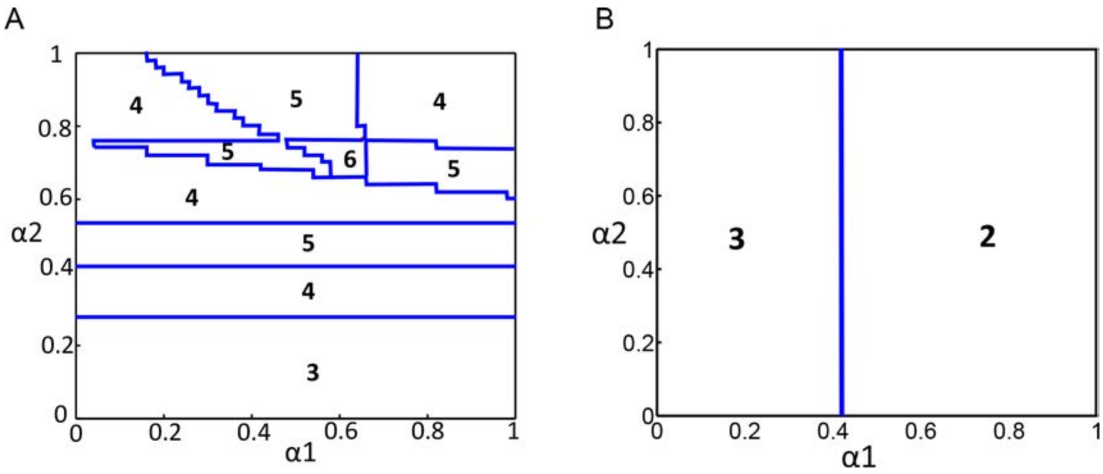

Supplementary Figure S2: State-space characteristics of the coupled EMT and stemness circuits at different SNAIL values. A. Phase diagram representing the number of stable states of the coupled circuit miR-200/ZEB/ LIN28/let-7 for different values of α1 and α2, such that the cells are in mesenchymal phenotype at no coupling (α1 = α2 = 0) as enabled by SNAIL = 240 * 10^3 molecules. **B.** Phase diagram representing the number of stable states of the coupled circuit miR-200/ZEB/ LIN28/let-7 for different values of α1 and α2, such that cells are in the epithelial phenotype at no coupling (α1 = α2 = 0) as enabled by SNAIL = 180 * 10^3 molecules. These levels of SNAIL are being determined from the analysis of (miR-200/ZEB) loop driven by SNAIL[4].

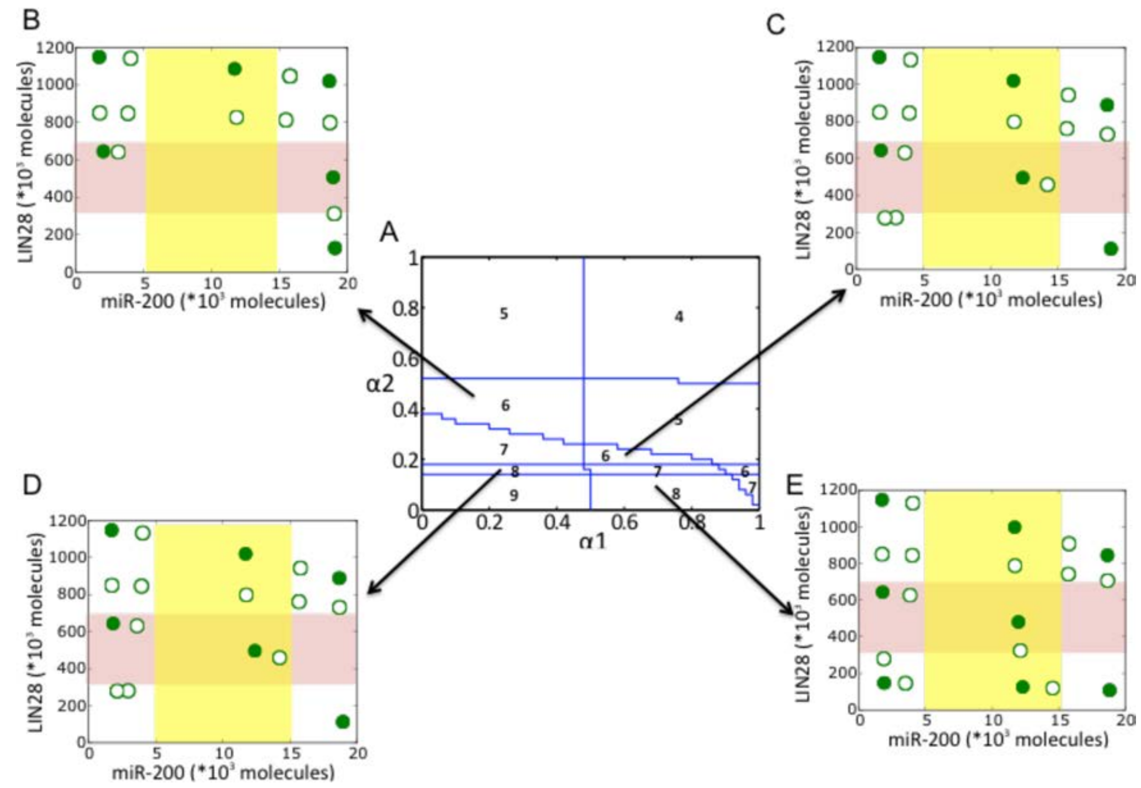

Supplementary Figure S3: State-space characteristics of the coupled EMT and stemness circuits – (miR-200/ZEB/LIN28/let-7) when cells are in the phase {E, E/M, M} at α1 = α2 = 0 (no coupling). A. Phase diagram representing the number of stable steady states of the coupled circuit (miR-200/ZEB/LIN28/let-7) for varying strengths of coupling (i.e. values of α1 and α2) under SNAIL = 200 * 10^3 molecules that enables the phase {E, E/M, M} at α1 = α2 = 0 (no coupling). **B.** Stable states of the coupled circuit at α1 = 0.3 and α2 = 0.4. **C.** Stable states of the coupled circuit at α1 = 0.6 and α2 = 0.2. **D.** Stable states of the coupled circuit at α1 = 0.3 and α2 = 0.16. **E.** Stable states of the coupled circuit at α1 = 0.7 and α2 = 0.1. Green solid dots represent stable states; green hollow dots are for unstable states. Red shaded rectangle defines the 'stemness window' based on relative OCT4 levels, and yellow shaded rectangle represents the range of miR-200 levels for the existence of the hybrid E/M phenotype, as identified by simulating the miR-200/ZEB circuit [4].

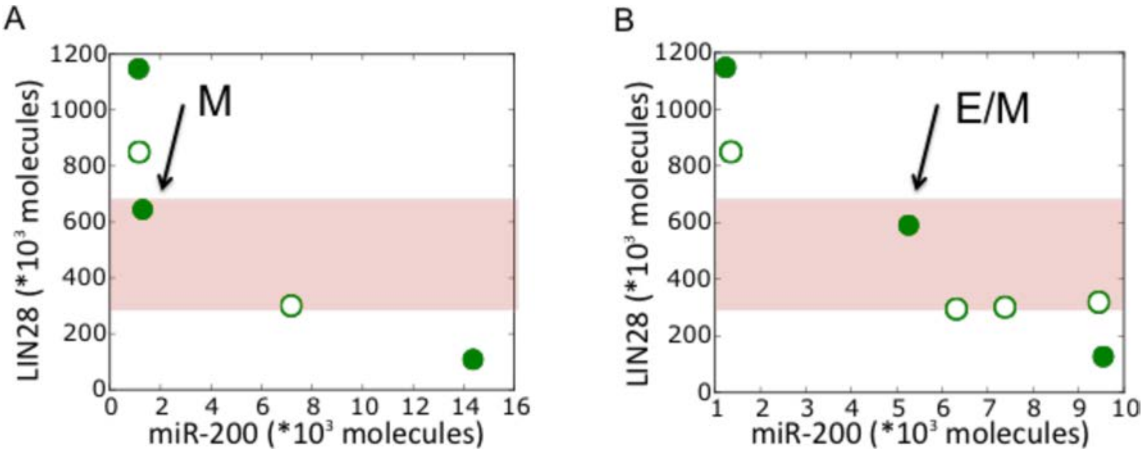

Supplementary Figure S4: State-space characteristics of coupled network miR-200/ZEB/LIN28/ let-7 and miR-200/ ZEB/LIN28/let-7/OVOL, when cells are in monostable {M} phase as enabled by SNAIL levels = 360 * 10^3 molecules at α1 = α2 = 0. A. Stable states of the coupled circuit without OVOL at α1 = α2 = 1 and driving signals SNAIL = 260 * 10^3 molecules and NF-kB = 25 * 10^3 molecules. Arrow highlights that the cells in mesenchymal phenotype can gain stemness. **B.** Stable states of the coupled circuit with OVOL at α1 = α2 = 1 and at driving signals SNAIL = 360 * 10^3 molecules and NF-kB = 25 * 10^3 molecules. Arrow highlights that the hybrid E/M phenotype can gain stemness. Red dashed rectangle defines the 'stemness window' based on relative OCT4 levels, and green dotted rectangle represents the range of miR-200 levels for the existence of the hybrid E/M phenotype.

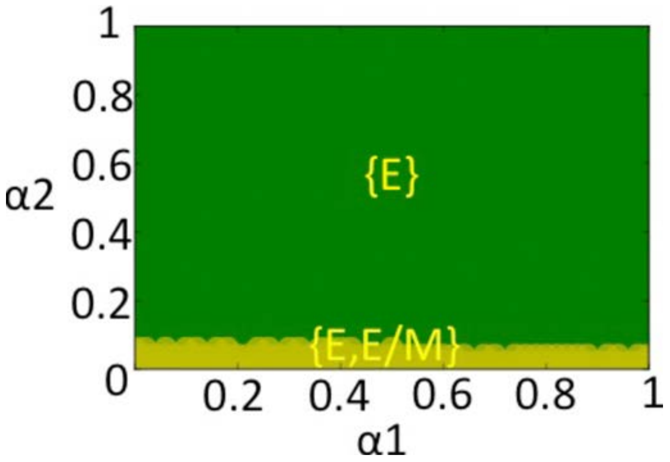

Supplementary Figure S5: State-space diagram of the circuit representing the values of (α1, α2) for which different phenotypes can attain stemness or lie in the 'stemness window' for the coupling circuit with OVOL and activation signal on OVOL under SNAIL levels = 300 * 10^3 molecules. Different colors represent the different combination of phenotypes that can gain stemness for a range of (α1, α2) values.

Supplementary Table S1: Parameters used for the production rates and degradation rates

Production rates (molecules/Hour)		Degradation rates ($Hour^{-1}$)	
$g_{\mu_{200}}$	2100	$k_{\mu_{200}}$	0.05
g_{m_Z}	11	k_{m_Z}	0.5
g_Z	100	k_Z	0.1
g_{μ_l}	200	k_{μ_l}	0.05
g_{m_L}	100	k_{m_L}	0.5
g_L	200	k_L	0.1
g_{m_O}	22	k_{m_O}	0.5
g_O	200	k_O	0.1

Supplementary Table S2: Parameters used in the shifted Hill functions

Weight factor		Threshold		Coefficient	
$\lambda_{Z,\mu_{200}}$	0.1	$Z^0_{\mu_{200}}$	220000	$n_{Z,\mu_{200}}$	3
$\lambda_{S,\mu_{200}}$	0.1	$S^0_{\mu_{200}}$	180000	$n_{S,\mu_{200}}$	2
λ_{Z,m_Z}	7.5	$Z^0_{m_Z}$	25000	n_{Z,m_Z}	2
λ_{S,m_z}	10	$S^0_{m_Z}$	180000	n_{S,m_Z}	2
α_{u_l,m_Z}	0~1	$\mu^0_{l_{m_z}}$	25000	n_{u_l,m_Z}	2
λ_{μ_l,μ_l}	11	$\mu_{l_{\mu_l}}$	12000	n_{μ_l,μ_l}	3
λ_{L,μ_l}	0.1	$L^0_{\mu_l}$	500000	n_{L,μ_l}	2
λ_{N,μ_l}	2	$N^0_{\mu_l}$	25000	n_{N,μ_l}	2
α_{u_{200},m_L}	0~1	μ^0_{200,m_L}	10000	n_{u_{200},m_L}	2
λ_{L,m_L}	3	$L^0_{m_L}$	200000	n_{L,m_L}	7
λ_{N,m_L}	2	$N^0_{m_L}$	250000	n_{N,m_L}	2
λ_{μ_l,m_L}	0.1	$\mu^0_{l_{m_L}}$	25000	n_{μ_l,m_L}	1
λ_{O,m_O}	0.1	$O^0_{m_O}$	25000	n_{O,m_O}	2
λ_{Z,m_O}	0.5	$Z^0_{m_O}$	10000	n_{Z,m_O}	1

Supplementary Table S3: Parameters in the L function (Hour^{-1})

l_0	l_1	l_2	l_3	l_4	l_5	l_6
1	0.6	0.3	0.1	0.05	0.05	0.05

Supplementary Table S4: parameters in the Y_m function (Hour^{-1})

γ_{m0}	γ_{m1}	γ_{m2}	γ_{m3}	γ_{m4}	γ_{m5}	γ_{m6}
0	0.04	0.2	1	1	1	1

Supplementary Table S5: parameters in the Y_μ function (Hour^{-1})

$\gamma_{\mu 0}$	$\gamma_{\mu 1}$	$\gamma_{\mu 2}$	$\gamma_{\mu 3}$	$\gamma_{\mu 4}$	$\gamma_{\mu 5}$	$\gamma_{\mu 6}$
0	0.005	0.05	0.5	0.5	0.5	0.5
